# Optimal Fenestration of the Fontan Circulation

**DOI:** 10.3389/fphys.2022.867995

**Published:** 2022-06-30

**Authors:** Zan Ahmad, Lynn H. Jin, Daniel J. Penny, Craig G. Rusin, Charles S. Peskin, Charles Puelz

**Affiliations:** ^1^ Courant Institute of Mathematical Sciences, New York University, New York, NY, United States; ^2^ School of Physics, Georgia Institute of Technology, Atlanta, GA, United States; ^3^ Department of Pediatrics, Section of Cardiology, Baylor College of Medicine and Texas Children’s Hospital, Houston, TX, United States

**Keywords:** Fontan circulation, compartmental model, hemodynamics, oxygen transport, fenestration

## Abstract

In this paper, we develop a pulsatile compartmental model of the Fontan circulation and use it to explore the effects of a fenestration added to this physiology. A fenestration is a shunt between the systemic and pulmonary veins that is added either at the time of Fontan conversion or at a later time for the treatment of complications. This shunt increases cardiac output and decreases systemic venous pressure. However, these hemodynamic benefits are achieved at the expense of a decrease in the arterial oxygen saturation. The model developed in this paper incorporates fenestration size as a parameter and describes both blood flow and oxygen transport. It is calibrated to clinical data from Fontan patients, and we use it to study the impact of a fenestration on several hemodynamic variables, including systemic oxygen availability, effective oxygen availability, and systemic venous pressure. In certain scenarios corresponding to high-risk Fontan physiology, we demonstrate the existence of a range of fenestration sizes in which the systemic oxygen availability remains relatively constant while the systemic venous pressure decreases.

## 1 Introduction

**FIGURE 1:**
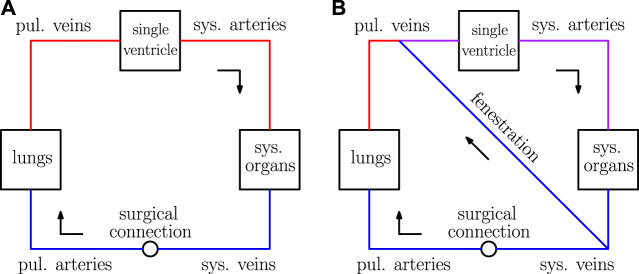
Sketches of the Fontan circulation in panel **(A)** and the fenestrated Fontan circulation in panel **(B)**. Blue represents deoxygenated blood, red represents oxygenated blood, and purple represents a mixture of oxygenated and deoxygenated blood.

Single ventricle physiology corresponds to a spectrum of congenital heart defects in which there is only one functioning ventricular chamber. Patients with this type of condition require complex medical and surgical interventions to ensure survival. The typical course of treatment for these defects is a sequence of surgeries during the first several years of life, ending with a procedure that establishes an abnormal physiology known as the Fontan circulation. This physiology was conceived in 1971 and is characterized by the systemic organs and lungs in series, as in a normal circulation (Fontan and Baudet, 1971). However, in contrast to a normal circulation, the Fontan physiology relies on passive blood flow to the lungs. This circulation is created by surgically placing the single functioning ventricle upstream from the systemic organs and connecting the vena cavae directly to the pulmonary arteries. Refer to the panel (A) of Figure 1 for a schematic of the Fontan circulation, where the label “surgical connection” corresponds to the connection established between the vena cavae and pulmonary arteries. It is important to note that this connection has relatively low resistance, resulting in a small difference between the systemic venous and pulmonary artery pressures.

Fontan patients may experience severe complications because of their abnormal physiology, including protein-losing enteropathy, ventricular and hepatic dysfunction, and plastic bronchitis (Feldt et al., 1996; Mondésert et al., 2013; Barracano et al., 2022). Many of these issues might be attributed to chronically low cardiac output because of the serialized organs and lungs leading to higher than normal systemic venous pressure (Rychik et al., 2012). The larger systemic venous pressure is certainly crucial for passively driving flow through the pulmonary vascular bed. Furthermore, the single ventricle is responsible for generating enough energy to push blood through both the systemic and pulmonary vasculature. The low resistance in the surgical connection between the pulmonary arteries and the vena cavae results in a pulmonary artery pressure that approximately equals the systemic venous pressure. In turn, this might lead to an elevated systemic venous pressure compared to that found in a normal circulation. Higher systemic venous pressure is thought to be a cause of complications (Rychik et al., 2012; Ohuchi, 2017). Theoretically, cardiac output can be increased while pulmonary artery and systemic venous pressures can be simultaneously decreased through the introduction of a shunt between the systemic veins and the pulmonary veins known as a fenestration.

In practice, this shunt is established between the systemic veins and atria as a “side-by-side” connection. In our model, the atria and venous compartments are lumped together, so the fenestration here is described by a shunt between the systemic and pulmonary veins. Furthermore, the nonlinear resistance model used for the fenestration in this paper is derived assuming two compartments are connected by a hole with a pre-determined cross-sectional area. This setup considered here is also consistent with the typical creation of such a connection. For a schematic of the Fontan circulation with a fenestration, refer to panel (B) of Figure 1.

A fenestration has been introduced into the Fontan physiology both at the time of Fontan conversion and also later in the patient’s life for the treatment of complications (Rychik et al., 1997; Lemler et al., 2002). While a fenestration typically increases cardiac output, it also decreases oxygen saturation of blood flowing to the systemic tissues. This is because blood flowing through the fenestration bypasses the lungs and is therefore not re-oxygenated. The trade-off between a decrease in oxygen saturation and an increase in cardiac output can also be seen in the Norwood physiology, which was mathematically studied using a non-pulsatile model by Barnea et al. (Barnea et al., 1994). This physiology is the result of the first surgery for the treatment of single ventricle disease. In the Norwood circulation, some of the blood exiting the single ventricle enters the pulmonary arteries while the rest enters the body. The authors demonstrated that the Norwood circulation can be “balanced” to optimize systemic oxygen availability to the tissues. This paper is concerned with similar questions regarding the Fontan physiology. We believe this study, focused on fenestrating the Fontan circulation, is of clinical relevance because of the aging Fontan population and possible therapeutic benefits of such an intervention (Rychik et al., 1997; Barracano et al., 2022).

The complexity of the Fontan circulation has motivated the development of many mathematical models that focus on various aspects of this physiology. For an extensive review of modeling efforts devoted to the Fontan physiology, see Degroff et al. (DeGroff, 2008). Researchers have created three dimensional fluid dynamics models for the connection between the vena cavae and pulmonary arteries, called the total cavopulmonary connection in an extracardiac Fontan circulation, in an effort to minimize energy loss in the surgical anatomy (Marsden et al., 2009; Yang et al., 2010). There has also been work using lower dimensional models that include one-dimensional or compartmental descriptions for fluid mechanics. Puelz et al. developed a one-dimensional/compartmental model of the Fontan physiology with a fenestration that was combined with a non-pulsatile description of oxygen transport (Puelz et al., 2017). This paper marks an improvement over their work by allowing for pulsatile oxygen transport, possible mixing in the systemic and pulmonary veins, and a more accurate description of the fenestration as a side-by-side connection rather than a conduit. Conover et al. used compartmental modeling to study the surgical stages for hypoplastic left heart syndrome, including the Fontan physiology (Conover et al., 2018). Liang et al. constructed compartmental models for both normal and Fontan circulations in order to systematically compare their hemodynamics (Liang et al., 2014). To our knowledge, the present paper describes one of the first models for the fenestrated Fontan circulation that employs a fully time-dependent description for both hemodynamics and oxygen transport.

We develop a pulsatile compartmental model of the Fontan physiology and use it to study the impact of a fenestration on blood flow and oxygen transport. The model, with the fenestration closed, is calibrated to clinical data in order to provide a baseline set of parameters. We vary both the pulmonary vascular resistance and fenestration size, with respect to the baseline model, to study the fenestration’s impact on several important variables, including systemic venous pressure, cardiac output, oxygen saturation, systemic oxygen availability, and effective oxygen availability. Our model demonstrates that in certain scenarios which correspond to high-risk Fontan physiology, a range of fenestration sizes exists in which the systemic oxygen availability remains relatively constant. In these cases, the systemic venous pressure decreases, which could be of substantial benefit to the patient.

## 2 Models and Methods

Our model for the Fontan circulation contains two main parts: 1) a blood flow model that incorporates a nonlinear resistance for the fenestration and 2) an oxygen transport model. The blood flow model is presented in the first subsection along with details for modeling the fenestration. The oxygen transport model is described in the second subsection. Methods for numerically approximating these models are detailed in the final subsection. The models and methods presented here closely follow those given in related work by Han et al., and additional details may be found in that paper (Han et al., 2021).

### 2.1 Blood Flow Model

The blood flow model used here describes the circulation as a series of compartments that are either compliance chambers or resistor elements. Our approach is derived from earlier work by Peskin and Tu, with modifications to account for the Fontan physiology and fenestration (Tu and Peskin, 1986; Tu and Peskin, 1989; Hoppensteadt and Peskin, 2013). Each chamber is identified with a unique index *i*. We assume the following relation between compliance *C*_*i*_, pressure *P*_*i*_, and volume *V*_*i*_:

$$V_{i}=V_{i,\text{d}}+C_{i}P_{i}.$$ (1)

The dead volume *V*_*i*,d_ is equal to the residual chamber volume at zero pressure. The heart chambers obey Equation 1, but in this case the compliance is taken to be a time-dependent function that varies periodically between minimum *C*_systole_ and maximum *C*_diastole_. Since our model describes single ventricle physiology, one side of the heart is removed from the circulation. The single ventricle in the Fontan circulation receives blood from the pulmonary veins and ejects that blood into the systemic arteries (refer to Figure 1). The single ventricle is a distinct chamber with a time-varying compliance and the atrium chambers are lumped in with the pulmonary venous chamber.

The time-dependent ventricular compliance, denoted *C*_ventricle_ = *C*_ventricle_(*t*), is taken to be a periodic function of time, with period *T* corresponding to the duration of the cardiac cycle. The compliance is defined as the reciprocal of the elastance *E*_ventricle_(*t*). To define the elastance function for the ventricle, we follow the approach from Mynard et al., except we modify their formula so that our elastance function is exactly periodic (Mynard et al., 2012). Specifically, the time-dependent equation for the elastance of the ventricle on the interval [0, *T*] is given by

$$E_{\text{ventricle}}\left(t\right)=k\;\frac{g_{1}\left(t\right)}{1+g_{1}\left(t\right)}\left(\frac{1}{1+g_{2}\left(t\right)}-\frac{1}{1+g_{2}\left(T\right)}\right)+E_{\text{min}}$$ (2)

where

$$g_{i}\left(t\right)={\left(\frac{t}{\tau_{i}}\right)}^{m_{i}},\;i=1,2.$$ (3)

The minimum and maximum elastances are *E*_max_ = 1/*C*_systole_ and *E*_min_ = 1/*C*_diastole_ and *k* is a normalization factor defined as follows:

$$k=\frac{E_{\text{max}}-E_{\text{min}}}{{\text{max}}_{t\in \left[0,T\right]}\left[\frac{g_{1}\left(t\right)}{1+g_{1}\left(t\right)}\left(\frac{1}{1+g_{2}\left(t\right)}-\frac{1}{1+g_{2}\left(T\right)}\right)\right]}.$$ (4)

The parameters *τ*_1_ and *τ*_2_ appearing in Equation 3 are the systolic and diastolic time constants, respectively. These values, along with *m*_1_ and *m*_2_, control the transition between the minimum and maximum elastance values for the single ventricle. In particular, larger values of *m*_1_ and *m*_2_ correspond to more rapid transitions between the minimum and maximum elastance values.

The resistor elements in our model describe connections between compliance chambers. In this framework, compliance chambers *i* and *j* have two connections between them. The connection from *i* to *j* contains resistance *R*_*ij*_ in series with a diode oriented from *i* to *j*. The connection from *j* to *i* is defined analogously. If *R*_*ij*_ = *R*_*ji*_, the compliance chambers are effectively connected by a single resistor and flow is allowed to move in both directions. To model a valve with no leak that allows flow from *i* to *j*, we set *R*_*ij*_ equal to the (typically small) resistance of the valve and *R*_*ji*_ equal to infinity. This setup, in which we separate flows in the two possible directions, makes it possible also to model leaky valves (by making both resistances finite but unequal), and it has further benefit in the simulation of oxygen transport. In practice, we work with the reciprocal of the resistance, i.e. the conductance, denoted *G*_*ij*_ = 1/*R*_*ij*_. The flow (volume per unit time) from chamber *i* to chamber *j*, denoted *Q* *_ij_*, is assumed to obey Ohm’s law,

$$Q_{ij}=S\left(P_{i},P_{j}\right)\;G_{ij}\;\left(P_{i}-P_{j}\right),$$ (5)

where *S*(*P*_*i*_, *P*_*j*_) is an indicator function that models the valve. This function equals one if *P*_*i*_ > *P*_*j*_ and zero otherwise.

The equations of motion for the blood flow model follow from conservation of volume in each compliance chamber. Using our notation given above and defining *N* to be the number of compliance chambers, we have

$$\frac{dV_{i}}{dt}=\overset{N}{\underset{j=1}{\sum }}\left(Q_{ji}-Q_{ij}\right),\;i=1,\ldots ,N.$$ (6)

Substituting Equations 1, 5 into (6), we obtain:

$$\frac{d}{dt}\left(C_{i}P_{i}\right)=\overset{N}{\underset{j=1}{\sum }}\left(S\left(P_{i},P_{j}\right)\;G_{ij}+S\left(P_{j},P_{i}\right)\;G_{ji}\right)\left(P_{j}-P_{i}\right),i=1,\ldots ,N.$$ (7)

This set of equations, one for each compliance chamber, comprise the blood flow model. Our model for Fontan circulation has four compliance chambers corresponding to the major vessel networks: the systemic arteries, systemic veins, pulmonary arteries, and pulmonary veins. The systemic organs, lungs, outflow valve, atrium/ventricle valve, and Fontan connection are taken to be resistance elements.

The fenestration between the systemic and pulmonary veins is taken to be a nonlinear resistor that depends on its cross-sectional area and the magnitude of flow through it. Let *Q*_Fe_ denote the fenestration flow and *A*_0_ denote its cross-sectional area. Let *P*_sv_ and *P*_pv_ denote the systemic venous and pulmonary venous pressures respectively. Positive fenestration flow corresponds to flow from the systemic venous chamber to the pulmonary venous chamber. Define the blood velocity through the fenestration to be *u* = *Q*_Fe_/*A*_0_ and the density of blood to be *ρ* = 1.06 g/cm^3^. For positive fenestration flow corresponding to *Q*_Fe_ > 0, Bernoulli’s equation describes the pressure drop from the systemic veins to the pulmonary veins in terms of the fluid velocity:

$$P_{\text{sv}}=P_{\text{pv}}-\frac{1}{2}\rho u^{2}=P_{\text{pv}}-\frac{\rho }{2A_{0}^{2}}Q_{\text{Fe}}^{2}.$$

For negative fenestration flow, we have the same equation as above, but with the pressures switched. These two cases, corresponding to either positive or negative fenestration flow, can be expressed in a single formula:

$$P_{\text{sv}}-P_{\text{pv}}=\frac{\rho }{2A_{0}^{2}}|Q_{\text{Fe}}|Q_{\text{Fe}}.$$ (8)

Equation 8 reveals the resistance of the fenestration, as a function of the magnitude of the fenestration flow and the size:

$$R_{\text{Fe}}=\frac{\rho }{2A_{0}^{2}}|Q_{\text{Fe}}|.$$ (9)

We add an additional term to this resistance, denoted *R*_visc_:

$$R_{\text{Fe}}=R_{\text{visc}}+\frac{\rho }{2A_{0}^{2}}|Q_{\text{Fe}}|.$$ (10)

This additional term physically corresponds to a small viscous resistance of the fenestration when the flow is close to zero. In our simulations, *R*_visc_ = 0.001 mmHg min L^−1^. It is also practically important since it allows for the fenestration conductance to remain finite when *Q*_Fe_ = 0. The formula for fenestration conductance is

$$G_{\text{Fe}}=\frac{1}{R_{\text{visc}}+\frac{\rho }{2A_{0}^{2}}|Q_{\text{Fe}}|},$$ (11)

which is used in (7) for the conductance between the systemic and pulmonary venous compliance chambers when the fenestration is open. Note that the equations in (7) are nonlinear because of the functions *S*(*P*_*i*_, *P*_*j*_). The fenestration conductance *G*_Fe_ introduces an additional nonlinearity which needs be carefully handled in the numerical method, to be described below.

### 2.2 Oxygen Model

In our model for oxygen transport, each compliance chamber has a time-dependent oxygen concentration denoted ${\left[O_{2}\right]}_{i}$ . This variable corresponds to a volumetric concentration so that $V_{i}{\left[O_{2}\right]}_{i}$ is the volume of oxygen in the *i*th compliance chamber. We also define sources and sinks of oxygen along the resistance elements between compliance chambers, denoted by the set of parameters *M*_*ij*_. These parameters are nonzero only for the connections between the arteries and veins on both sides of the circulation. Conservation of oxygen in the circulation model is described by the following set of equations:

$$\frac{d}{dt}\left(V_{i}{\left[{\text{O}}_{2}\right]}_{i}\right)=\overset{N}{\underset{\begin{array}{c} j=1\\ j\neq i \end{array}}{\sum }}\left(Q_{ji}{\left[{\text{O}}_{2}\right]}_{j}-Q_{ij}{\left[{\text{O}}_{2}\right]}_{i}+M_{ji}\right)\;i=1,\ldots ,N.$$ (12)

The first term in the sum on the right hand side is the rate of oxygen entering chamber *i* from *j*, using the concentration from chamber *j*. The second term is the rate of oxygen leaving chamber *i*, and the third term, if nonzero, is either a source (*M*_*ji*_ > 0) or sink (*M*_*ji*_ < 0). For the connection between the systemic arteries and systemic veins, the baseline value of oxygen consumption, denoted *M*_sa,sv_, is determined from Fick’s law, assuming the concentration in the systemic veins is 60% of the concentration in the systemic arteries (Hijazi et al., 1992):

$$M_{\text{sa,sv}}=-0.4\;Q_{\text{s}}\;\gamma .$$ (13)

In computing *M*_sa,sv_ from Equation 13, *Q*_s_ is taken to be the cardiac output from our model Fontan circulation without the fenestration, and *γ* corresponds to the volumetric oxygen concentration in blood when it is fully saturated. The source of oxygen from the lungs, denoted *M*_pa,pv_, is chosen in a time-dependent fashion so that the blood flowing into the pulmonary veins from the pulmonary arteries is fully saturated. This requirement implies the following equation for *M*_pa,pv_:

$$M_{\text{pa,pv}}=Q_{\text{p}}\left(\gamma -{\left[{\text{O}}_{2}\right]}_{\text{pa}}\right),$$ (14)

where *Q*_p_ is the pulmonary flow and ${\left[O_{2}\right]}_{\text{pa}}$ is the oxygen concentration in the pulmonary arteries. This source term ensures that blood flowing into the pulmonary veins from the pulmonary arteries has a maximum volumetric oxygen concentration of *γ* = 0.201. This concentration is calculated by assuming an oxygen-binding capacity for hemoglobin (Hb) of 1.34 ml oxygen per g Hb and that 15 g Hb is in 100 ml of blood. This implies a maximum oxygen content of 20.1 ml oxygen per 100 ml of blood, corresponding to a maximum volumetric oxygen concentration of 0.201 (Pittman, 2016). In the presence of a fenestration, however, this stream of fully oxygenated blood mixes with the systemic venous blood that arrives in the pulmonary venous compartment via the fenestration, with the result that blood in the pulmonary venous compartment is not fully saturated.

### 2.3 Numerical Methods

In this section, we describe the numerical approximation schemes for the blood flow and oxygen transport models. Let *n* denote the time set size index and *δt* > 0 denote the time step. For the blood flow equations, we use the backward Euler scheme:

$$\frac{C_{i}^{n}P_{i}^{n}-C_{i}^{n-1}P_{i}^{n-1}}{\delta t}=\overset{N}{\underset{j=1}{\sum }}\left(S\left(P_{i}^{n},P_{j}^{n}\right)G_{ij}^{n}+S\left(P_{j}^{n},P_{i}^{n}\right)G_{ji}^{n}\right)\left(P_{j}^{n}-P_{i}^{n}\right).$$ (15)

There are two sources of nonlinearity in these equations: the valve state function *S*, which depends on the pressures, and the fenestration conductance, which depends on the flow. Since the valve state function *S* can only take on the values 0 or 1, our approach for solving the nonlinear system Equation 15 is to guess the value for each *S* variable, to solve the resulting linear system for the pressures, and finally to check whether the pressure values obtained are consistent with the assumed values of *S*. If there is any inconsistency, we change the state of those *S* values that were inconsistent with the pressures and try again. This is repeated until the pressures and all of the valve states have stopped changing, i.e., until the nonlinear system Equation 15 has been solved. Our initial guess for this process is taken to be values at the previous time step, which for almost all time steps is the correct choice. To deal with the nonlinearity in the fenestration conductance, we perform a fixed-point iteration on the fenestration flow, with the flow at the previous time step used as the initial guess. This iteration helps to avoid non-physical oscillations in the calculated fenestration flow waveform. Our numerical discretization for the oxygen transport equations is the following:

$$\frac{{\left[O_{2}\right]}_{i}^{n}V_{i}^{n}-{\left[O_{2}\right]}_{i}^{n-1}V_{i}^{n-1}}{\delta t}=\overset{N}{\underset{\underset{j\neq 1}{j=1}}{\sum }}\left({\left[O_{2}\right]}_{j}^{n-1}Q_{ji}^{n}-{\left[O_{2}\right]}_{i}^{n-1}Q_{ij}^{n}+M_{ji}\right).$$ (16)

The scheme is forward Euler in terms of the concentrations. We use the flow values calculated at the next time step since we have them available via Ohm’s law after updating the pressures with Equation 15.

## 3 Results and Discussion

In this section, we present and discuss results from our numerical simulations. As mentioned in Subsection 2.3, 10 fixed point iterations per time step are performed on Equation 15 to avoid nonphysical oscillations caused by the nonlinear fenestration conductance. The duration of the cardiac cycle is set to *T* = 0.016 min and is determined by stroke volume and cardiac output data from Fontan patients reported by Liang et al. (Liang et al., 2014) (see Table 3). In particular, we calculated this parameter using a stroke volume index of 0.04 L m^−2^ and a cardiac index of 2.5 L min^−1^ m^−2^. As a result of the model calibration process (to be described below) which determines dead volumes, compliances, and initial pressures, the total blood volume used in all simulations is 5 L. Initial oxygen concentrations in all compliance chambers are set to *γ* = 0.201 L of oxygen per L of blood. The choice of initial oxygen concentrations has no effect on the periodic steady state that is eventually reached. Mean values of variables reported below correspond to an average taken over the last three cardiac cycles of the simulation.

### 3.1 Model Calibration With a Closed Fenestration

We first describe the calibration of our model using hemodynamic data from Fontan patients, with the goal of deriving a baseline set of parameters. In this case, we use 1,000 time steps per cardiac cycle, and simulations are run for 40 cardiac cycles. This duration is long enough to ensure periodic steady states are reached for all hemodynamic variables, which are needed for the model calibration. The model is calibrated, with the fenestration closed, to a data set provided by Liang et al. (Liang et al., 2014). Tables 1, 2 display the parameters used within the calibrated model. Table 3 shows clinical variables calculated from our calibrated model compared to those reported by Liang et al. (Liang et al., 2014). Calibration was done by manual tuning with normal circulation parameters used as initial guesses. The duration of the cardiac cycle is fixed to 0.016 min for the model calibration procedure. Variables that incorporate blood volume are indexed to a body surface area of 1.5 m^2^. We remark that the outflow valve resistance is important for generating a gradual transition from isovolumetric contraction to ventricular ejection. This resistance parameter was chosen so that the peak pressure gradient between the ventricle and aorta during the open state of the valve is approximately 8 mmHg, as suggested in Mynard et al. (Mynard et al., 2012). Furthermore, the value for the outflow valve resistance used here is close to that used for the non-stenotic aortic valve case in Wisneski et al. (Wisneski et al., 2020). In general, variables from our baseline model match well with the clinical data.

**TABLE 1:** Parameters for the circulation model. Abbreviations: s, systemic organs; p, pulmonary; ov, outflow valve; av, atrium/ventricle valve; fo, Fontan connection; sa, systemic arteries; pa, pulmonary arteries; sv, systemic veins; pv, pulmonary veins.

Parameters	Resistance (*R*)	Dead Volume (*V*_d_)	Compliance (*C*)
Units	mmHg min L^−1^	L	L mmHg^−1^
s	20.78	—	—
p	0.5517	—	—
ov	0.20	—	—
av	0.01	—	—
fo	0.01	—	—
sa	—	0.7051	7.333 × 10^–4^
pa	—	0.0930	0.00412
sv	—	2.869	0.0990
pv	—	0.1475	0.01

**TABLE 2:** Parameters for the time varying ventricular compliance in the heart model.

Parameters	Symbol	Units	Ventricle
Minimal elastance	*E*_min_	mmHg L^−1^	79.52
Maximal elastance	*E*_max_	mmHg L^−1^	5,232
systolic exponent	*m*_1_	—	1.32
diastolic exponent	*m*_2_	—	27.4
Systolic time constant	*τ*_1_	min	0.269 × *T*
Diastolic time constant	*τ*_2_	min	0.452 × *T*
Dead volume	*V*_d_	L	0.028
Period of heartbeat	*T*	min	0.016

**TABLE 3:** Hemodynamic variables calculated from our calibrated model with a closed fenestration, compared to the clinical data reported by Liang et al. (Liang et al., 2014). Based on a report by Ohuchi, our model’s vena cava mean pressure, cardiac index, end diastolic volume index, and ejection fraction are all within the range of a “late-surviving” and “excellent-surviving” Fontan patient (Ohuchi, 2017). The indexing in the first four variables assumes a body surface area of 1.5 m^2^.

Variable	Our model	Clinical data reported in (Liang et al., 2014)
cardiac index (L min^−1^ m^−2^)	2.683	2.9, 2.1
stroke volume index (ml m^−2^)	42.93	39,40
end diastolic volume index (ml m^−2^)	75.91	72, 76
end systolic volume index (ml m^−2^)	32.98	33, 36
end systolic pressure (mmHg)	111.8	—
end diastolic pressure (mmHg)	6.828	6.6
vena cava mean pressure (mmHg)	9.347	8
pulse pressure (mmHg)	56.52	54
systemic artery systolic pressure (mmHg)	118.2	124
systemic artery diastolic pressure (mmHg)	61.68	70
systemic artery mean pressure (mmHg)	92.99	88
pulmonary artery mean pressure (mmHg)	9.306	9

**FIGURE 2:**
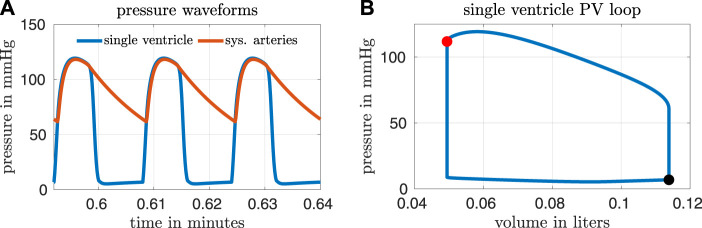
Results from our calibrated model with a closed fenestration. Three pressure waveforms from the end of the simulation are shown in panel **(A)** and a volume loop for the single ventricle is shown in panel **(B)**. The end-systolic and end-diastolic parts of the pressure-volume loop are indicated by the red and black markers respectively.

Based on a report by Ohuchi, the calibrated variables from our model correspond to a “late-surviving” and “excellent-surviving” Fontan patient (Ohuchi, 2017). “Late-surviving” refers to a follow-up of at least 15 years after the Fontan operation, and “excellent-surviving” is characterized by a central venous pressure of 10 mmHg, a cardiac index of 2.6 L min^−1^ m^−2^, an end diastolic volume index of 70 ml m^−2^, and an ejection fraction of 55% (refer to Table 1 in (Ohuchi, 2017)). These hemodynamic values align well with those from our calibrated model. Figure 2 shows results from our calibrated model with a closed fenestration. The left panel depicts pressure waveforms for the ventricle and systemic arteries over three cardiac cycles. The right figure shows the ventricular pressure-volume loop.

### 3.2 Blood and Oxygen Transport With an Open Fenestration

In this section, we explore the effect of an open fenestration on both hemodyamic and oxygen transport variables. In these cases, we use 100 time steps per cardiac cycle and simulations are run for 4,000 cardiac cycles. This duration is long enough to ensure periodic steady states are reached for all hemodynamic and oxygen transport variables. As described in Subsection 2.2, the baseline value for oxygen consumption in our models is calculated from Fick’s law via Equation 13, assuming 60% saturation in the systemic veins and using the systemic flow *Q*_s_ from the calibrated model with the closed fenestration. These choices result in a baseline oxygen consumption value of *M*_sa,sv_ = −0.3236 L min^−1^. The opening of the fenestration alters flow through the systemic and pulmonary beds, and in practice their corresponding resistances might change in response to these changes in flow. However, all parameters, except the fenestration size, remain fixed for the following experiments. This modeling choice stems from the hypothesis that Fontan patients have impaired responsiveness of their vascular beds to changes in flow (Ciliberti et al., 2012). For example, Ridderbos et al. identified pulmonary vascular remodeling in Fontan patients and concluded that such changes might impact pulmonary vasodilation (RidderbosRidderbos et al., 2015). Future work will incorporate variable systemic and pulmonary resistance parameters if clinical data is available and indicates this possibility. To separately address possible physiologic changes in parameters as a result of the fenestration, Subsection 3.3 describes a sensitivity analysis of our models.

**FIGURE 3:**
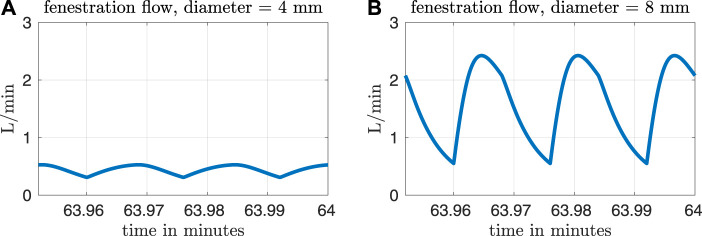
Fenestration flow waveforms for two different fenestration sizes and our baseline pulmonary vascular resistance value of *R*_p_ = 0.5517 mmHg min L^−1^. The waveform in panel **(A)** corresponds to a fenestration with diameter equal to 4 mm, and the waveform in panel **(B)** corresponds to a diameter of 8 mm.

Figure 3 shows fenestration flow waveforms for two different fenestration sizes. The left panel corresponds to a fenestration with diameter equal to 4 mm and the right panel corresponds to a diameter of 8 mm. The pulmonary vascular resistance is set to our baseline value of *R*_p_ = 0.5517 mmHg min L^−1^. Although the model allows for bi-directional flows, the fenestration flow throughout the cardiac cycle is exclusively from the systemic veins to the pulmonary veins, with more flow occurring in the larger fenestration. Note that the fenestration flow is roughly proportional to the cross-sectional area of the fenestration, since it is approximately multiplied by four when the diameter of the fenestration approximately doubles. This makes sense, since flow proportional to area means that velocity is constant, as we would expect from the Bernoulli relation for a given pressure difference. At larger fenestration sizes, however, the pressure difference would be expected to diminish, and the flow would eventually saturate at some level that is independent of the fenestration size.

**FIGURE 4:**
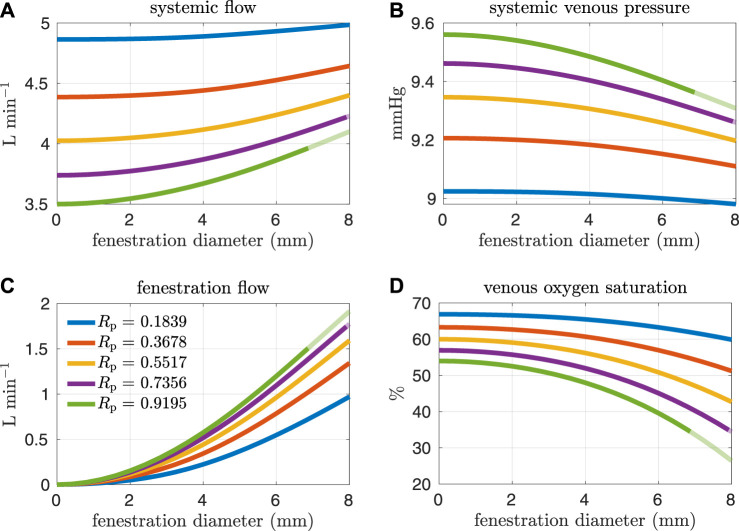
Results corresponding to oxygen consumption equal to −0.3236 L min^−1^. Pulmonary resistance values *R*_p_ centered around our baseline value are considered. Mean values for different hemodynamic and oxygen transport variables are plotted as functions of the fenestration diameter. **(A)** systemic flow, **(B)** systemic venous pressure, **(C)** fenestration flow, **(D)** venous oxygen saturation.

**FIGURE 5:**
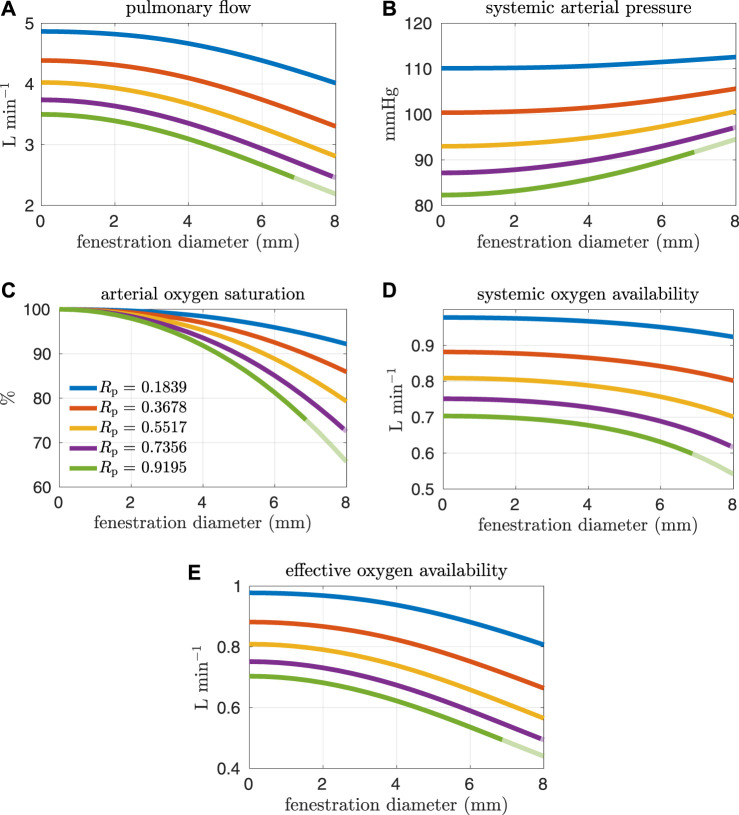
Results corresponding to oxygen consumption equal to −0.3236 L min^−1^. Pulmonary resistance values *R*_p_ centered around our baseline value are considered. Mean values for different hemodynamic and oxygen transport variables are plotted as functions of the fenestration diameter. Note that both the systemic oxygen availability and effective oxygen availability curves are monotonically decreasing as the fenestration diameter increases. **(A)** pulmonary flow, **(B)** systemic arterial pressure, **(C)** arterial oxygen saturation, **(D)** systemic oxygen availability, **(E)** effective oxygen availability.

Figures 4, 5 show results from our models with the oxygen consumption parameter *M*_sa,sv_ set to the baseline value. Sections of some curves are opaque and refer to fenestration sizes that result in systemic venous saturations less than 35%. To explore the effect of pulmonary resistance on hemodynamic and oxygen transport variables, we consider five values for the resistance that are equally spaced around the baseline value of *R*_p_ = 0.5517 mmHg min L^−1^. This range of values overlaps with pulmonary vascular resistances measured clinically in Fontan patients, e.g. the interquartile range 0.733–1.33 mmHg min L^−1^ for the pulmonary vascular resistance of adults without a failing Fontan reported by Sallmon et al. (adjusting for a body surface area of 1.5 m^2^) (Sallmon et al., 2021). All variables are plotted as functions of the fenestration diameter. The range of fenestration diameters considered here, from 0–8 mm, contains the range of diameters seen in clinical reports, see e.g. (Grosse-Wortmann et al., 2013; Vyas et al., 2007; Rychik et al., 1997). Figures 4A,B show systemic flow and systemic venous pressure. As expected, an increase in fenestration size leads to an increase in systemic flow and a decrease in systemic venous pressure. Changes in these variables are more pronounced for higher pulmonary vascular resistances. Figures 4C,D show the mean flow through the fenestration and the systemic venous oxygen saturation. In general, fenestration flow is larger for higher pulmonary vascular resistances, and it increases monotonically with fenestration diameter. The values for mean fenestration flow from our models are physiologically reasonable when compared to flows measured in patients via phase contrast cardiac magnetic resonance imaging (Grosse-Wortmann et al., 2013). Blood flowing through the fenestration is not reoxygenated, so larger fenestration sizes and pulmonary vascular resistances result in lower systemic venous saturations. Note that the systemic venous oxygen saturation for the closed fenestration and for the case *R*_p_ = 0.5517 mmHg min L^−1^ is 60%, which is precisely how the baseline oxygen consumption parameter for this set of experiments was determined. Another reason we examine systemic venous saturation is to ensure it is positive for a given set of parameters, since there is nothing in the model to preclude this variable from going negative. Figures 5A,B show pulmonary flow and systemic arterial pressure respectively. As expected, pulmonary flow decreases for increasing fenestration flow, which corresponds to increasing fenestration size. The overall increase in the cardiac output for larger fenestration sizes leads to an increase in the systemic arterial pressure. Figure 5C-E show systemic arterial oxygen saturation, systemic oxygen availability, and effective oxygen availability. Systemic availability is defined as the product of systemic flow and arterial oxygen saturation, see e.g. Barnea et al. (Barnea et al., 1994). As suggested by Koeken et al., effective oxygen availability is an important variable to consider in the presence of shunts (Koeken et al., 2012). Since blood through the fenestration is not reoxygenated, the effective availability disregards this shunt flow. It is defined with respect to stream of blood which is reoxygenated, i.e. the product of the pulmonary flow and the maximum volumetric oxygen concentration. The trends in systemic arterial oxygen saturation are the same as for the systemic venous saturation. For systemic oxygen availability, all curves monotonically decrease with increasing fenestration diameter. The trends for effective oxygen availability are similar; this variable decreases as the fenestration size increases. This is because larger fenestration flow leads to smaller pulmonary flow, resulting in a decrease in effective oxygen availability. A more dramatic decrease in systemic oxygen availability can be seen for larger pulmonary resistances. The monotonic nature of the systemic oxygen availability curve changes when we consider parameter values corresponding to a “high-risk” Fontan patient, to be described below.

**FIGURE 6:**
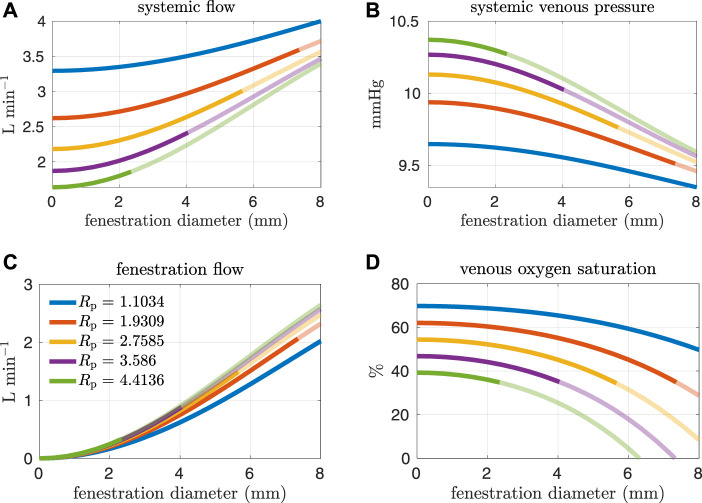
Results corresponding to oxygen consumption equal to −0.2 L min^−1^. Pulmonary resistance values *R*_p_ larger than our baseline value are considered. Mean values for different hemodynamic and oxygen transport variables are plotted as functions of the fenestration diameter. **(A)** systemic flow, **(B)** systemic venous pressure, **(C)** fenestration flow, **(D)** venous oxygen saturation.

**FIGURE 7:**
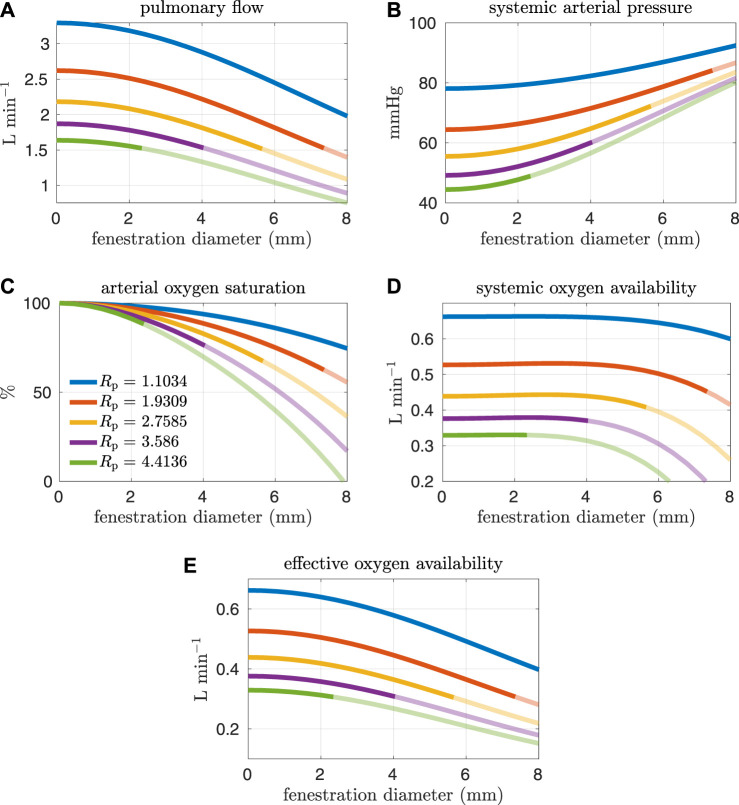
Results corresponding to oxygen consumption equal to −0.2 L min^−1^. Pulmonary resistance values *R*_p_ larger than our baseline value are considered. Mean values for different hemodynamic and oxygen transport variables are plotted as functions of the fenestration diameter. Note that the systemic oxygen availability curves achieve an optimal value for a nonzero fenestration size. However, the effective oxygen availability, which is important for quantifying the supply of oxygen, monotonically decreases as the fenestration size increases. **(A)** pulmonary flow, **(B)** systemic arterial pressure, **(C)** arterial oxygen saturation, **(D)** systemic oxygen availability, **(E)** effective oxygen availability.

High pulmonary vascular resistance and low cardiac output have been identified as important risk factors for Fontan patients (Lemler et al., 2002; Egbe et al., 2017). More specifically, a pulmonary vascular resistance index^1^ (PVRI) greater than 2 mmHg min L^−1^ m^2^ and a cardiac index less than 2.5 L min^−1^ m^−2^ are criteria that have been used to characterize high-risk patients (Egbe et al., 2017). Referring to Table 3, the PVRI and cardiac index for our baseline model are 3.465 mmHg min L^−1^ m^2^ and 2.686 L min^−1^ m^−2^ respectively. According to Egbe et al., our baseline model aligns with the cohort containing more favorable Fontan physiology. In the next set of experiments, we consider pulmonary vascular resistance values that are larger than our baseline value in order to generate models that correspond to “high-risk” Fontan physiology. The resistance values considered here are close to the interquartile range for Fontan patients reported in Agnoletti et al. (1.33–3.13 mmHg min L^−1^) when considering a body surface area of 1.5 m^2^ (Agnoletti et al., 2017). Figures 6, 7 and show analogous results to Figures 4, 5 except for pulmonary resistance values larger than our baseline value. Note that an increase in pulmonary vascular resistance substantially decreases the cardiac output. In these cases, we set the oxygen consumption parameter to *M*_sa,sv_ = −0.2 L min^−1^ so that venous saturations remain in a reasonable range. Otherwise, the drop in cardiac output, with a fixed oxygen consumption, would lead to small and/or negative systemic venous saturations for a large range of fenestration sizes. This parameter value was chosen with reference to the clinical data reported in Hijazi et al., see e.g. patient 6 (Hijazi et al., 1992).

In general, the fenestration has a more substantial impact on hemodynamics and oxygen transport for larger pulmonary vascular resistances. The trends seen here are similar to those in Figures 4, 5, except for systemic oxygen availability. In these high-risk cases (with larger pulmonary vascular resistance, smaller cardiac index, and smaller oxygen consumption), the systemic oxygen availability curve is relatively constant, instead of decreasing, for an initial range of fenestration sizes. The increase in cardiac output, as a result of the open fenestration, is able to compensate for the decrease in systemic arterial oxygen saturation, at least for small enough fenestration diameters. These results point towards the possibility of fenestrating high-risk patients to achieve lower pulmonary artery/systemic venous pressures and higher cardiac output without the cost of a decrease in systemic oxygen availability. Note that the fenestration flow, which is larger in these cases, seems to increase more slowly for larger fenestration sizes. This trend might be a result of the nonlinear fenestration resistance, which is larger for larger flows. The inflection point coincides with a very modest peak in systemic oxygen availability, as mentioned above. However, the effective oxygen availability, the variable important for describing oxygen transport, monotonically decreases with the fenestration diameter. While a fenestration will result in higher systemic flows, it will not result in a better supply of oxygen. A fenestration might only benefit patients because systemic perfusion and blood pressure might be easier to maintain in exercise, while systemic venous pressure will be lower than in the non-fenestrated case.

The effect of the fenestration on hemodynamic and oxygen transport variables, as predicted by our models, is consistent with trends seen in clinical case reports (Rychik et al., 1997; Vyas et al., 2007). Vyas et al. and Rychik et al. report on patients that were given a fenestration for the mitigation of complications. Their papers include some clinical data collected both pre- and post-fenestration. According to their data, the fenestration increases cardiac output and decreases arterial saturation. These changes are also predicted by our models. Although the clinical data is somewhat variable, the magnitude of these changes for single-defect fenestrations, which range in size from 4–8 mm, are close to what is seen in our models. A limitation of our approach is the lack of precise calibration to a pre-fenestration data set in order to perform model validation with post-fenestration data. In future work, it will be necessary to procure data for this purpose.

### 3.3 Sensitivity Analysis

The previous section considered variations in several hemodynamic and oxygen transport variables as functions of the fenestration size, pulmonary vascular resistance, and oxygen consumption parameters. In reality, the introduction of a fenestration may induce physiologic changes in many of the parameters in the model. To quantify the impact these changes might have on important variables, we perform a sensitivity analysis in three distinct cases: 1) with our baseline model corresponding to *R*_p_ = 0.5517 mmHg min L^−1^ and a closed fenestration, 2) with *R*_p_ = 0.5517 mmHg min L^−1^ and a fenestration diameter of 4 mm, and 3) with *R*_p_ = 2.7585 mmHg min L^−1^ and a fenestration diameter of 4 mm. Cases 1 and 2 use an oxygen consumption value of *M*_sa,sv_ = −0.3236 L min^−1^ and case 3 uses a value of *M*_sa,sv_ = −0.2 L min^−1^. Since a theme of this paper is the impact of the fenestration on systemic venous pressure, systemic oxygen availability, and effective oxygen availability, we consider these three variables in the analysis that follows.

For a variable *g* = *g*(*X*) as a function of a parameter *X*, the sensitivity with respect to the parameter *X*, evaluated at a nomimal value *X*_0_, is defined to be:

$${\left.\frac{X_{0}}{g\left(X_{0}\right)}\frac{\partial g}{\partial X}\right|}_{X=X_{0}}.$$

We approximate the sensitivity numerically by using a forward difference approximation to the partial derivative:

$${\left.\frac{X_{0}}{g\left(X_{0}\right)}\frac{\partial g}{\partial X}\right|}_{X=X_{0}}\approx \frac{X_{0}}{g\left(X_{0}\right)}\frac{g\left(X\right)-g\left(X_{0}\right)}{X-X_{0}}.$$

In the above formula, *X* is taken to be 1.1 *X*_0_, i.e. a ten percent perturbation in the nominal parameter *X*_0_. For each of the three cases, we first compute results from a single simulation with the nominal parameter values. Then, we perform a sequence of simulations in which each parameter is independently increased to ten percent above its nominal value. We use the same numerical setup as described in Subsection 3.2.

Tables 4–6 show sensitivities for the systemic venous pressure, systemic oxygen availability and the effective oxygen availability with respect to the main model parameters. Each row refers to one of the three cases described above. The most significant parameter for determining the systemic venous pressure is the compliance of the systemic veins. The next largest sensitivities include those with respect to the other compliances, as well as those corresponding to the systemic and pulmonary resistances. In case 3, corresponding to an open fenestration and larger pulmonary resistance, the sensitivity of the systemic venous pressure with respect to the systemic venous compliance increases relative to cases 1 and 2. The sign of the sensitivities for the compliance parameters are negative, indicating (at least locally around the nominal value) that an increase in compliance leads to a decrease in systemic venous pressure.

**TABLE 4:** Sensitivities for cases 1, 2, and 3 computed for the systemic venous pressure.

	*R*_s_	*R*_p_	*R*_av_	*R*_ov_	*R*_fo_	*C*_sa_
Case 1	−5.6e-02	3.9e-02	4.5e-04	−2.2e-04	8.7e-04	−5.8e-02
Case 2	−5.7e-02	3.4e-02	3.9e-04	−2.2e-04	7.6e-04	−5.9e-02
Case 3	−4.4e-02	3.8e-02	9.0e-06	−1.8e-04	2.1e-04	−4.0e-02

	*C*_pa_	*C*_sv_	*C*_pv_	*E*_min_	*E*_max_	
Case 1	−3.3e-02	−7.4e-01	−6.4e-02	5.0e-02	7.3e-03
Case 2	−3.3e-02	−7.4e-01	−6.7e-02	5.3e-02	7.1e-03
Case 3	−3.5e-02	−7.9e-01	−4.5e-02	4.2e-03	1.3e-02

**TABLE 5:** Sensitivities for cases 1, 2, and 3 computed for the systemic oxygen availability.

	*R*_s_	*R*_p_	*R*_av_	*R*_ov_	*R*_fo_	*C*_sa_
Case 1	−2.1e-01	−2.3e-01	−3.7e-03	−1.1e-03	−4.1e-03	−3.1e-02
Case 2	−2.2e-01	−2.5e-01	−4.5e-03	−1.2e-03	−4.4e-03	−3.4e-02
Case 3	−1.7e-01	−5.6e-01	−3.0e-03	−9.3e-04	−2.0e-03	−2.7e-02

	*C*_pa_	*C*_sv_	*C*_pv_	*E*_min_	*E*_max_	
Case 1	−3.3e-02	−7.4e-01	−4.7e-02	−6.1e-01	1.8e-01
Case 2	−3.5e-02	−7.9e-01	−5.9e-02	−6.7e-01	2.0e-01
Case 3	−4.3e-02	−9.7e-01	−8.3e-03	−5.2e-01	1.5e-01

**TABLE 6:** Sensitivities for cases 1, 2, and 3 computed for the effective oxygen availability.

	*R*_s_	*R*_p_	*R*_av_	*R*_ov_	*R*_fo_	*C*_sa_
Case 1	−2.1e-01	−2.3e-01	−3.7e-03	−1.1e-03	−4.1e-03	−3.1e-02
Case 2	−2.2e-01	−2.9e-01	−5.7e-03	−1.2e-03	−5.2e-03	−3.3e-02
Case 3	−1.5e-01	−5.7e-01	−3.4e-03	−8.2e-04	−2.1e-03	−2.4e-02

	*C*_pa_	*C*_sv_	*C*_pv_	*E*_min_	*E*_max_	
Case 1	−3.3e-02	−7.4e-01	−4.7e-02	−6.1e-01	1.8e-01	
Case 2	−3.4e-02	−7.7e-01	−7.4e-02	−6.5e-01	2.0e-01	
Case 3	−3.8e-02	−8.5e-01	−1.4e-02	−4.6e-01	1.4e-01	

In all cases, the systemic oxygen availability and effective oxygen availability also appear to be most sensitive to the systemic venous compliance. Note that for case 1, the systemic and effective oxygen availabilities and their corresponding sensitivities are equal since the shunt flow is zero. Both variables are very sensitive to the systemic and pulmonary resistances, likely due to the significant impact of these parameters on cardiac output. The sensitivities with respect to the minimum and maximum elastances of the single ventricle are also notable. Notice that the sensitivities of the oxygen availabilities with respect to *E*_min_ are negative, while the sensitivities with respect to *E*_max_ are positive. These signs are consistent with the notion that a stiffer chamber in diastole (corresponding to larger *E*_min_) experiences less diastolic filling, leading to smaller cardiac output and availability. In contrast, stronger chamber contraction (corresponding to larger *E*_max_) leads to an increase cardiac output, pulmonary flow, and oxygen availability.

In summary, the sensitivity analysis of our models indicates the importance of the systemic and pulmonary resistances as well as the single ventricle contraction parameters on oxygen availabilities and the systemic venous pressure. Although care was taken to calibrate the model with a closed fenestration to a clinical data set from Fontan patients, results for the open fenestration cases will depend on the chosen parameters. The pulmonary vascular resistance has been cited as important parameter in determining Fontan hemodynamics (Ciliberti et al., 2012), and its significance is demonstrated in our sensitivity analysis for the systemic and effective oxygen availabilities. For this reason, we focused on varying this parameter in order to quantify its impact across a range of fenestration sizes. General trends predicted from our models in arterial oxygen saturation and cardiac output, between the closed and open fenestration cases, are consistent with clinical case reports (Rychik et al., 1997; Vyas et al., 2007). To our knowledge, most papers exploring the fenestration intervention do not have the necessary pre-fenestration data for accurate model calibration. In turn, a limitation of our models is the lack of precise calibration to a pre-fenestration data set in order to predict post-fenestration hemodynamics, for the purpose of model validation. Future work will entail the collection of this data and the patient-specific calibration of the model to Fontan patients, in order to predict the fenestration’s impact on their hemodynamics.

## 4 Conclusion

In this paper, we developed a pulsatile compartmental model of the Fontan circulation that describes blood flow and oxygen transport. It also incorporates a fenestration between the systemic and pulmonary veins. The model was calibrated to clinical data with the fenestration closed in order to create a baseline set of parameters.

We then studied the impact of an open fenestration on several hemodynamic and oxygen transport variables. An open fenestration decreased the pulmonary artery pressure and increased cardiac output, with larger impacts on these variables seen for larger values of the pulmonary vascular resistance. For our baseline oxygen consumption value and for pulmonary vascular resistances close to the baseline value, systemic oxygen availability monotonically decreased as a function of the fenestration diameter. For pulmonary vascular resistances that are typical of at-risk patients, however, the systemic oxygen availability for small fenestration sizes remained relatively constant until a critical size was reached, at which point the availability dropped quickly. This trend was accompanied by a decrease in systemic venous (≈ pulmonary arterial) pressure. Furthermore, as the fenestration size increased, the effective oxygen availability decreased in all cases. Thus, the reason for fenestrating the Fontan circulation may not so much be to affect systemic or effective oxygen availability, but rather to ensure that systemic oxygen availability is not reduced by an intervention with a different benefit–the reduction of systemic venous pressure.

## Data Availability

The original contributions presented in the study are included in the article, further inquiries can be directed to the corresponding author.

## Footnotes

1 PVRI calculated here does not directly correspond to the *R*_p_ parameter in our models. In Egbe et al., it is defined as the mean pulmonary artery pressure divided by the cardiac index (Egbe et al., 2017), while the typical definition would be the pressure gradient across the lungs divided by the pulmonary flow index. Note that for a closed fenestration, the pulmonary flow index and cardiac index are equal.
